# In Vitro Biodegradation, Drug Absorption, and Physical Properties of Gelatin–Fucoidan Microspheres Made of Subcritical-Water-Modified Fish Gelatin

**DOI:** 10.3390/md21050287

**Published:** 2023-05-03

**Authors:** Truc Cong Ho, Ju-Sop Lim, Shin-Jun Kim, Sung-Yeoul Kim, Byung-Soo Chun

**Affiliations:** 1 PL MICROMED Co., Ltd., 1F, 15-5, Yangju 3-gil, Yangsan-si 50620, Republic of Korea; hctruc357@gmail.com (T.C.H.); casseur1@naver.com (J.-S.L.); sinxovnd1@naver.com (S.-J.K.); kim41409114@gmail.com (S.-Y.K.); 2 Department of Food Science and Technology, Pukyong National University, 45 Yongso-ro, Nam-gu, Busan 48513, Republic of Korea

**Keywords:** subcritical water hydrolysis, fish gelatin, fucoidan, microspheres, physical properties, biodegradation, drug absorption

## Abstract

This study aimed to prepare gelatin–fucoidan microspheres with enhanced doxorubicin binding efficiency and controllable biodegradation using fish gelatin combined with low molecular weight (LMW) gelatin and fucoidan at fixed ratios. The MW of gelatin was modified by subcritical water (SW), which is known as a safe solvent, at 120 °C, 140 °C, and 160 °C. In addition, gelatin–fucoidan microspheres were prepared using a solvent exchange technique. Our findings revealed that particle size decreased, the surface was rougher, the swelling ratio increased, and particle shape was irregular in microspheres composed of SW-modified gelatin. Doxorubicin binding efficiency was improved by fucoidan and SW-modified gelatin at 120 °C but not at 140 °C and 160 °C. Interestingly, an increase in in vitro enzymatic degradation was observed in the microspheres consisting of SW-modified fish gelatin, although the cross-linking degree between them was not significantly different. This is because LMW gelatin could form more cross-linked bonds, which might be weaker than the intramolecular bonds of gelatin molecules. Gelatin–fucoidan microspheres consisting of SW-modified fish gelatin with controlled biodegradation rates could be a candidate for a short-term transient embolization agent. In addition, SW would be a promising method to modify the MW of gelatin for medical applications.

## 1. Introduction

Recently, microspheres have been used intensively as promising drug delivery systems and embolization agents. Microspheres can be made from various materials such as polysaccharides (cellulose and chitosan), proteins (albumin and collagen), and other polymers, including poly(lactic acid) and poly(glycolic acid) [1]. Although the materials to prepare microspheres are diverse, they should be biodegradable and biocompatible to minimize the side effects. Among promising sources for producing microspheres, gelatin is a potential candidate. 

Gelatin is a partially hydrolyzed product of fibrous protein collagen extracted from animal skin, bone, and connective tissue. Gelatin is most commonly used in food due to its unique properties, such as chewiness, texture, foam stabilization, creaminess, fat reduction, mouthfeel, emulsification, gelling, and water-binding [2]. In medical applications, gelatin has been applied in tissue engineering, wound healing, and bone generation [3,4,5]. In the pharmaceutical field, gelatin is a novel drug delivery agent since it has intrinsic features that enable the design of carrier systems such as microparticles and nanoparticles [6]. Kang et al. [7] developed a doxorubicin (DOX)–fish gelatin nano gel system with high DOX-loading efficiency that effectively inhibits the growth of NIH3T3 cells. Conjugation between epigallocatechin gallate and gelatin-DOX-coated gold nanoparticles for fluorescence imaging and growth inhibition of prostate cancer cells has been studied [8]. A system of DOX-gelatin-coated gold nanoparticles responding to different pH and temperature conditions has also been reported recently [9]. In addition, protein–polysaccharide complexes showed better functional properties than the proteins and polysaccharides alone [10]. In that context, gelatin/chitosan microspheres were prepared for drug release [11], and fucoidan–gelatin microparticles with posaconazole for antifungal activity were also investigated [12]. 

Low molecular weight (LMW) gelatin can effectively improve physical conditions and is promising in terms of application in the food field. LMW gelatin can be obtained by enzymatic [13] and chemical [14] processes. However, the enzymatic method is time-consuming, whereas the chemical method uses more toxic chemicals that might harm humans and the environment. Recently, subcritical water (SW) has been considered a ‘green’ and effective solvent to extract and modify MW of biopolymers [15,16]. Thus, SW could be a promising intermediate technique to regulate the MW of gelatin for further applications. 

Fucoidan, mainly obtained from brown seaweed, is a polysaccharide containing many L-fucose and sulfate ester groups in its molecules. Many studies on the applications of this marine polysaccharide have been carried out due to its excellent biofunctional properties such as antitumor, anti-inflammatory, anticoagulant and antithrombotic, antivirus, blood–lipid reducing, antioxidant activities, uropathy, and renal pathology, gastric protective effects and therapeutic potential in surgery [17]. Particularly, fucoidan with LMW has a good anticancer capacity [18]. With its significant biological activities, fucoidan has high potential use in medical and pharmaceutical fields. In drug delivery applications, recently, DOX-loaded fucoidan-capped gold nanoparticles have been examined [19]. The conjugate of fucoidan-oleic acid for the delivery of curcumin and paclitaxel was also established [20]. Lu et al. [21] developed a new type of multifunctional fucoidan-based nanoparticles to inhibit MDA-MB-468 cancer cells. In addition, fucoidan is a negative polysaccharide due to SO^–3^ groups in its molecule structure. Thus, it can induce the drug binding efficiency with drugs containing negative charges such as Doxorubicin. 

Microspheres used for permanent embolization might obstruct non-target blood vessels or produce certain side effects. On the other hand, depending on the stages of the disease, transient embolization, which lasts from minutes to months, could reduce liver damage and facilitate avoidance of ischemia-induced neovascularization [22]. Thus, it is essential to control the degradation rate of gelatin microspheres to optimize the treatment. It is believed that microspheres made from a mixture of fish gelatin, LMW gelatin, and fucoidan could control their biodegradation rates and improve doxorubicin binding efficiency. Therefore, this study aimed to produce LMW gelatin using SW as an environmentally friendly technique. Changes in physical properties such as MW, color, viscosity, and antioxidant activity of the gelatin hydrolysates were investigated to confirm the benefits of this technique. Gelatin-based microspheres consisting of only fish gelatin; fish gelatin and fucoidan; fish gelatin, fucoidan, and SW-modified gelatin were prepared using a solvent exchange technique. Then, the prepared microspheres were investigated regarding physical properties, degradation rates, and drug-binding efficiency.

## 2. Results

### 2.1. Changes in Physical Properties of SW-Modified Gelatin Hydrolysates

#### 2.1.1. Color, pH, and Viscosity

The influences of SW on the physical characteristics of SW-modified gelatin hydrolysates are presented in Table 1. The color of gelatin hydrolysates decreased with the increase in SW temperatures from 120 °C to 160 °C. Notably, the color intensity sharply decreased when gelatin was treated at 160 °C. Gelatin may contain lipids, sugars, and other proteins [23]. Therefore, the increase in darkness of the hydrolysates might result from the Maillard reaction [24] rather than the thermal decomposition of protein to form furfural, an intermediate product of the browning process [25]. The pH values were not considerably changed among the hydrolysates. In the range of temperatures from 120 °C to 160 °C, the self-ionization of water and degradation of glycine (melting point at 233 °C) might not occur, and therefore pH of hydrolysates was stable. In a previous study, pH values of collagen hydrolysates rapidly increased when temperatures increased from 220 °C to 250 °C due to the decomposition of glycine to form methylamine and other amines [24]. The changes in physical properties of the hydrolysates were also confirmed by the viscosity, which sharply decreased after treatment with SW at 120 °C (1.6 cP) compared to fish gelatin (7.18 cP) at the same concentration. However, the viscosity slightly declined when temperatures increased from 120 °C to 160 °C. These results confirmed that there might be changes in gelatin MW after treatment in SW from 120 °C to 160 °C.

#### 2.1.2. MW of SW-Modified Gelatin

Gel permeation chromatography was employed to determine the MW of the fish gelatin and SW-modified gelatin, and results are presented in Table 2. After treatment in SW at 120 °C, the MW of gelatin decreased more than twofold compared to the fish gelatin (12.2 and 26 kDa, respectively). When the temperature increased to 140 °C, threefold reduction in gelatin MW was observed (from 12.2 to 4.1 kDa). However, gelatin MW slightly decreased when the temperature was raised to 160 °C. In this study, CO_2_ gas was used to pressurize the water during the SW hydrolysis process, forming a weak acidic condition when a high temperature was applied. Because the solubility of CO_2_ increases in the subcritical region of water, the acid-catalyzed reaction pathway might be induced significantly [26], leading to a sharp decrease in gelatin MW. In another study, total amino acid concentration in HCl hydrolysate after treatment at 160 °C and 25 °C was similar, indicating no significant degradation in amino acid compositions after treatment at 160 °C [27]. These authors observed that the thermal degradation of amino acids was seen at temperatures from 180 °C to 240 °C. In addition to CO_2_, we also used nitrogen as a pressurized gas, and the outcomes showed that nitrogen gas had less effect on the decrease in gelatin MW compared to CO_2_ (data are not shown here because the comparison of the effects of two gases is out of the scope of this study). Our previous study also observed the effects of nitrogen gas in SW condition on protein MW [28]. The obtained results from the present study indicated that SW from 120 °C to 160 °C and pressurized by CO_2_ can be used to lessen MW of gelatin. 

### 2.2. Antioxidant Activity of Fish Gelatin and SW-Modified Gelatin Hydrolysates

**Figure 1:**
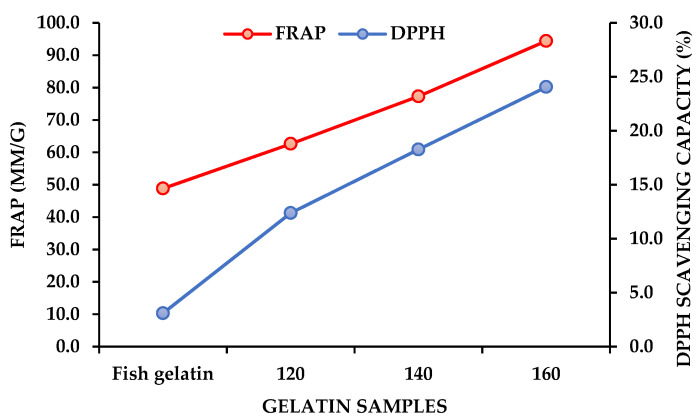
Antioxidant activity of fish gelatin and SW-modified gelatin hydrolysates. 120—SW-modified gelatin hydrolysates at 120 °C, 140—SW-modified gelatin hydrolysates at 140 °C, and 160—SW-modified gelatin hydrolysates at 160 °C.

Effects of SWH on antioxidant activities (DPPH and FRAP) of SW-modified gelatin were also investigated, presented in Figure 1. The increasing trend in antioxidant activity was noticeably perceived when the temperatures of SW raised from 120 °C to 160 °C and compared to fish gelatin. These results are also consistent with those of our previous work when Comb Pen Shell hydrolysates performed an increasing trend in their antioxidant activity after being treated by SW from 120 °C to 220 °C [28]. The highest DPPH scavenging (24%) and FRAP (94.4 mM Trolox/g) detected in the hydrolysate treated at 160 °C is probably due to the formation of free amino acids and peptides which are known as scavenging and antioxidant compounds. This high biological activity of SW-modified gelatin was also contributed by some antioxidant compounds produced by the Maillard reaction.

### 2.3. Fourier-Transform Infrared Spectroscopy

**Figure 2:**
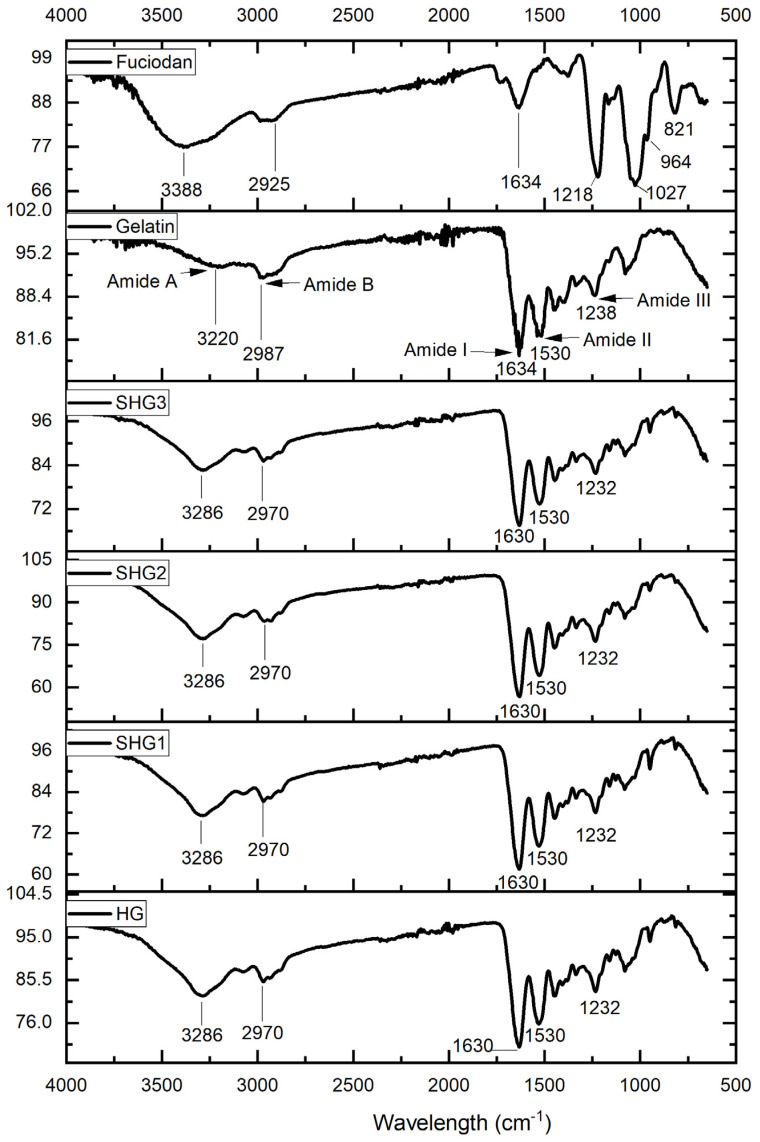
FTIR spectra of fish gelatin, fucoidan, and gelatin–fucoidan microspheres. HG—microspheres made by fish gelatin and fucoidan; SHG1—microspheres made by fish gelatin, SW-modified gelatin at 120 °C, and fucoidan; SHG2—microspheres made by fish gelatin, SW-modified gelatin at 140 °C, and fucoidan; and SHG3—microspheres made by fish gelatin, SW-modified gelatin at 160 °C, and fucoidan.

To confirm the interaction between gelatin and fucoidan in the prepared microspheres, the FTIR of pure components is also presented in Figure 2. The broad band at 3388 cm^−1^ in fucoidan spectra is assigned to the hydrogen bond (O–H) group, commonly seen in all polysaccharides’ structures. This band was more dominant than the amide A band of pure gelatin at approximately 3220 cm^−1^ when combined in the spectra of all microspheres. A weak peak with a center ranging from 2900 cm^−1^ to 3000 cm^−1^ in fucoidan spectra is typically for the C–H stretching vibration, whereas the peak at approximately 2987 cm^−1^ in gelatin spectra is representative of amide B, which was also observed in all spectra of microspheres. Three strong signals at 1634 cm^−1^, 1530 cm^−1^, and 1238 cm^−1^ were observed in gelatin and all microsphere spectra, which were assigned to amide I, amide II, and amide III of protein structure, respectively. A strong peak seen at 1025 cm^−1^ is typically for the stretching vibration of sulphoxides (S = O) of the fucoidan molecule structure. The other two peaks at 964 cm^−1^ and 821 cm^−1^ were assigned to the asymmetrical and symmetrical stretching vibrations of C–O–S bonds of sulfate groups, which were also observed in the spectra of all microspheres.

### 2.4. Scanning Electron Microscopy (SEM)

**Figure 3:**
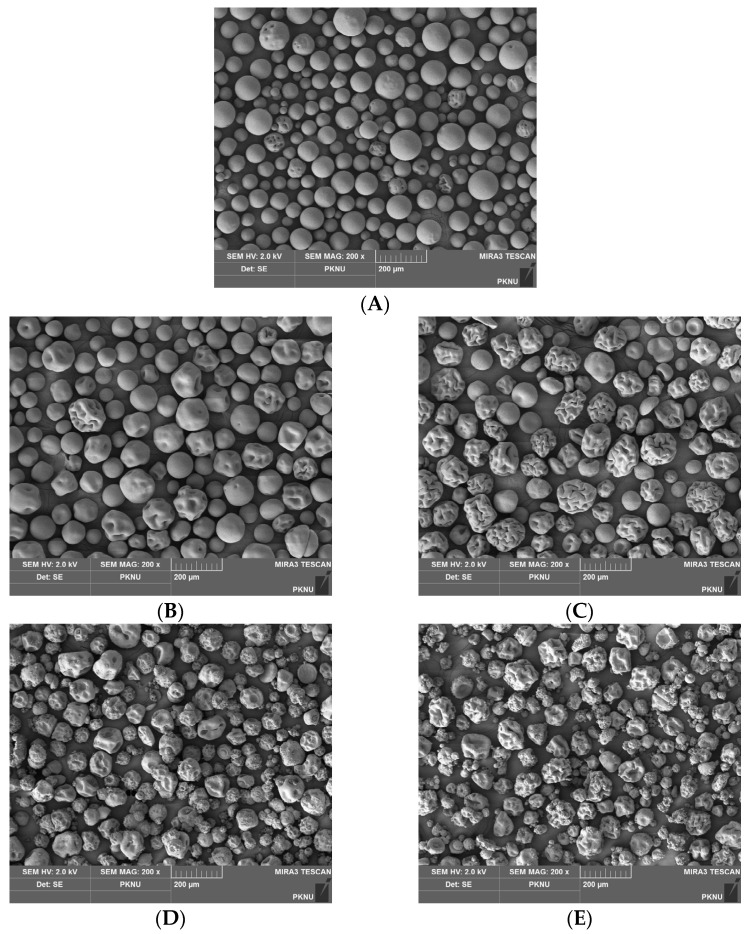
Surface morphology of microspheres. Control—microspheres made by fish gelatin only (**A**); HG—microspheres made by fish gelatin and fucoidan (**B**); SHG1—microspheres made by fish gelatin, SW-modified gelatin at 120 °C, and fucoidan (**C**); SHG2—microspheres made by fish gelatin, SW-modified gelatin at 140 °C, and fucoidan (**D**); and SHG3—microspheres made by fish gelatin, SW-modified gelatin at 160 °C, and fucoidan (**E**).

The surface morphology of microspheres was investigated using scanning electron microscopy (SEM), presented in Figure 3. It can be seen that most of the control microspheres, those made of gelatin only (Figure 3A), had a smooth and spherical shape, whereas HG microspheres (Figure 3B) appeared with some porosity. The particle sizes of both HG and SHG1 (Figure 3C) are relatively similar. However, SHG1 microspheres were more porous than the HG ones. The porosity formation in all microspheres probably occurs because they contain some LMW gelatin, and it might occur in the solvent exchange step. Briefly, when alcohol is added to the water-in-oil emulsion, water in droplets is gradually replaced, and biopolymers tend to shrink when they come into contact with alcohol. Considering one droplet contains high MW and LMW gelatin molecules, these molecules rearrange, and porosities are formed in a location of LMW gelatin molecules. Porosity formation can also be induced in the cross-linking step where gelatin molecules are closer after cross-linked with a zero cross-linker, EDC. A similar trend was also seen in microspheres SHG2 (Figure 3D) and SHG3 (Figure 3E), which have a rough surface. These two microspheres are tiny because they were made from a combination of fish gelatin and SW-modified gelatin at 140 °C and 160 °C with MW of approximately 4.1 and 2.8 kDa, respectively. It can also be seen that the particle sizes of these two microspheres are ununiform. This might be due to the unequal distribution of high MW and LMW gelatin molecules in droplets, although their ratio was similar in the water phase (1:1 ratio). The un-spherical shape observed in HG, SHG1, SHG2, and SHG3 microspheres might be due to fucoidan (MW of approximately 8 kDa), which might react in the same manner with LMW gelatin during the solvent exchange step. 

### 2.5. Cross-Linking Degree of Gelatin–Fucoidan Microspheres

**Figure 4:**
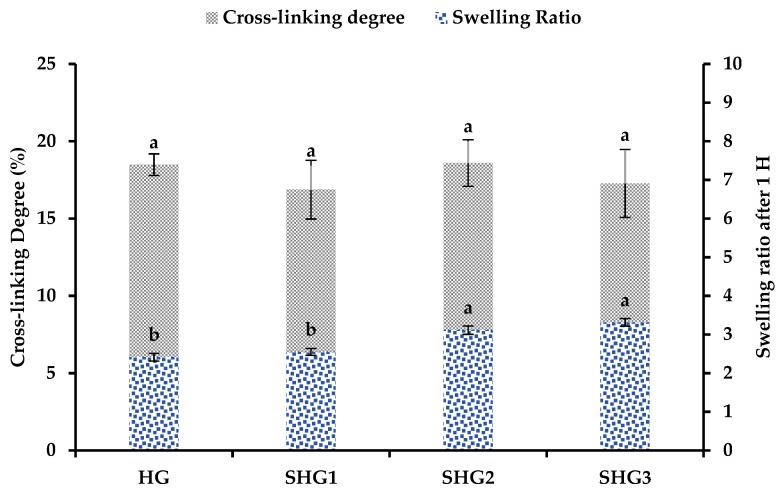
Cross-linking degrees and swelling property of microspheres. HG—microspheres made by fish gelatin and fucoidan; SHG1—microspheres made by fish gelatin, SW-modified gelatin at 120 °C, and fucoidan; SHG2—microspheres made by fish gelatin, SW-modified gelatin at 140 °C, and fucoidan; and SHG3—microspheres made by fish gelatin, SW-modified gelatin at 160 °C, and fucoidan. Different letters in each type of chart indicate significant differences (*p* < 0.05).

EDC is known as a zero cross-linker and is widely used to cross-link proteins or chitosan molecules through amine groups in their structures. In this study, the microspheres were cross-linked with EDC; the results are presented in Figure 4. It can be seen that all microspheres had no significant difference in their degrees of cross-linking with approximate value of 18%. The cross-linking degree of the microspheres is lower than that obtained in a previous study, 53.8% [29], which used only gelatin and cross-linked under the same condition. Fucoidan might prevent several gelatin molecules from standing close to each other, leading to weak interactions between gelatin molecules or groups of gelatin molecules. Regarding swelling properties, it can be seen that SHG3 was swollen the most after soaking in PB pH 7.4, whereas the opposite result was obtained in the HG. Cross-linked bonds that EDC formed between amine groups of gelatin molecules might be weaker than their intracellular bonds. When microspheres containing many cross-linked bonds contact an aqueous solution, they tend to increase their size more quickly.

### 2.6. In Vitro Enzymatic Degradation of Microspheres

**Figure 5:**
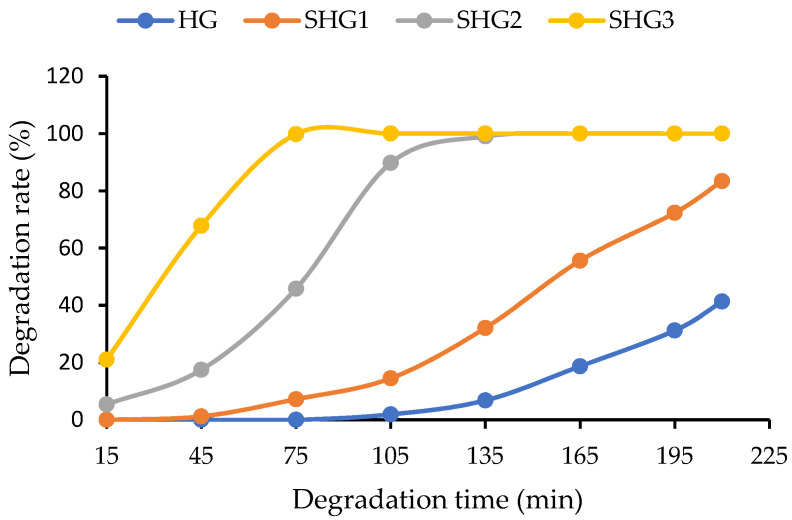
Enzymatic degradation of microspheres in 0.1% pepsin in 0.1 N HCl. HG—microspheres made by fish gelatin and fucoidan; SHG1—microspheres made by fish gelatin, SW-modified gelatin at 120 °C, and fucoidan; SHG2—microspheres made by fish gelatin, SW-modified gelatin at 140 °C, and fucoidan; and SHG3—microspheres made by fish gelatin, SW-modified gelatin at 160 °C, and fucoidan.

Through enzymatic hydrolysis in the body, gelatin microspheres are degraded by proteolytic enzymes such as trypsin, collagenase, and pepsin [30]. In the present study, 50 mg microspheres were soaked in 1.8 mL pepsin solution (0.1% in 0.1 N HCl) and incubated at 37 °C, and the results were obtained (Figure 5). The SHG3 microspheres were degraded the most after the first 15 min, and they were utterly digested by pepsin after 75 min. SHG2 could extend its stability for less than 135 min in a pepsin solution, whereas more than 10% of SHG1 microspheres remained after 210 min. HG, which contains only the fish gelatin, was relatively stable, with nearly 60% of microspheres remaining after this time. Biodegradation rates between microspheres made from gelatin with different MW were different, although they had similar cross-linking degrees. The microspheres composed of more LMW gelatin have more cross-linked bonds between amine groups, which can be easily degraded when exposed to the proteolytic enzyme compared to intramolecular bonds. The microspheres with quick biodegradation might be suitable for a short-term transient embolic agent.

### 2.7. Doxorubicin Absorption Capacity

**Figure 6:**
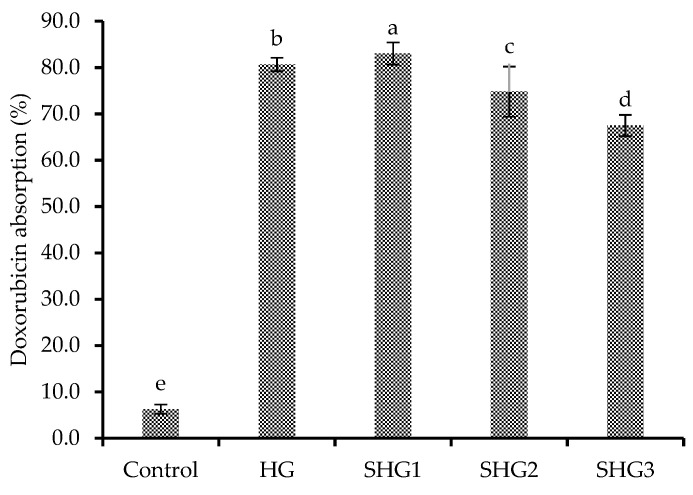
Doxorubicin absorption capacity of microspheres. Control—microspheres made of only fish gelatin; HG—microspheres made by fish gelatin and fucoidan; SHG1—microspheres made by fish gelatin, SW-modified gelatin at 120 °C, and fucoidan; SHG2—microspheres made by fish gelatin, SW-modified gelatin at 140 °C, and fucoidan; and SHG3—microspheres made by fish gelatin, SW-modified gelatin at 160 °C, and fucoidan. Different letters indicate the significant difference (*p* < 0.05).

Both fish gelatin and doxorubicin have positive charges in their molecule structures; therefore, the binding efficiency of microspheres with the drug might not be significant. Therefore, this study used fucoidan with negative charges of SO^3−^ groups to enhance drug binding capacity. As seen in Figure 6, the control (microspheres without fucoidan) absorbed a meager amount of the drug, less than 10%. On the other hand, the remaining microspheres are significantly bound with the drug. The highest drug binding efficiency was detected in SHG1 microspheres, with a value of approximately 83%, as they have more porosity on the surface than the others (Figure 3). However, the efficiency decreased in the microspheres consisting of fish gelatin combined with SW-modified gelatin at 140 °C and 160 °C (SHG2 and SHG3) by approximately 75% and 67.5%, respectively. Drug binding efficiency might depend on the electrostatic interaction between fucoidan and doxorubicin and the pore size of the microspheres. When microspheres (SHG2 and SHG3) were over-swollen, their pore size increased significantly, which might not significantly entrap the drug inside. On the contrary, those with a mild swelling ratio might have moderate pore sizes (HG and SHG1) and could effectively keep the drug inside their structures.

## 3. Materials and Methods

### 3.1. Materials

Fish gelatin (type A and 250 Bloom) was purchased from Geltech (Busan, Republic of Korea). Fucoidan was obtained from MSC Fucoidan (Seoul, Republic of Korea). Paraffin oil, isopropyl alcohol, and Sorbitan Monooleate (Span80) were attained from Duksan Company (Gyeonggi, Republic of Korea). Pepsin 1:10,000 derived from porcine stomach mucosa was bought from FUJIFILM Wako Pure Chemical (Osaka, Japan). 1-Ethyl-3-(3-dimethyl aminopropyl) carbodiimide was acquired from Daejung (Busan, Republic of Korea). Other chemicals were of analytical grade. 

### 3.2. Subcritical Water Modification of Gelatin

**Figure 7:**
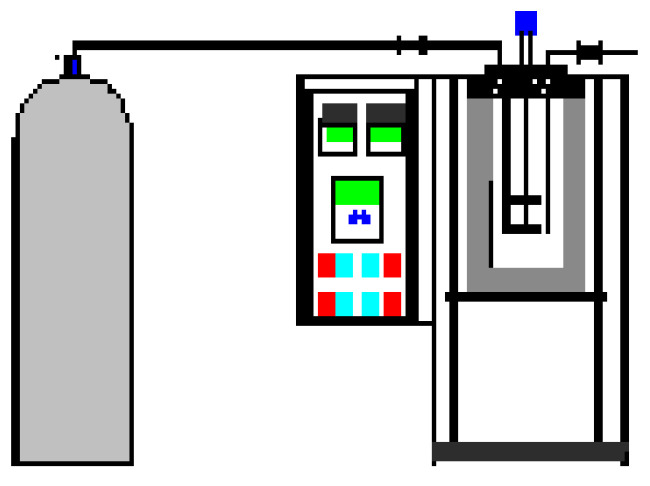
Flow diagram of the subcritical water hydrolysis system.

Fish gelatin and distilled water were introduced into a 1000 mL reactor of the SWH system from Phosentech Co., Ltd (Daejeon, Republic of Korea) (Figure 7). Pressure, hydrolysis time, and stirring speed were fixed at 50 bar, 30 min, and 200 rpm. Three temperatures of SW used in this study were 120 °C, 140 °C, and 160 °C. The heating time to obtain the target temperatures was approximately 50 min. After finishing the reaction time, the mixture was cooled to 65 °C with tap water through a stainless steel pipe inside the reactor. The pressure was slowly released when the mixture temperature was lower than 100 °C. Physical properties of SW-modified gelatin, such as viscosity, pH, and color, were determined before being lyophilized and stored at −20 °C for further use. 

### 3.3. Viscosity, pH, and Color Measurement

The viscosity of SW-modified gelatin hydrolysates was measured using a Brookfield Model DV-II+ (USA). In this measurement, a fixed volume of gelatin hydrolysates (5 mL) was added to the temperature control jacket cup, which was heated by a water bath to obtain the target temperature of 25 °C. The spindle S140 was employed with a speed of 12 rpm. The measurement was conducted in triplicate. For color measurement, the Cielab color space of the hydrolysates was determined using a reflectance colorimeter (Lovibond^®^ RT series, Tintometer Ltd., Amesbury, UK). The results were reported in three values, L *, a *, and b *, representing lightness, green-red, and blue–yellow, respectively. The values of pH were determined at 25 °C and repeated three times. 

### 3.4. Gel Permeation Chromatography 

MW of fish gelatin and SW-modified gelatin were measured according to a study previously reported [31] through a gel permeation chromatography system (Dionex Ultimate 3000, Sunnyvale, CA, USA) equipped with an Ultrahydrogel column and an RI detector. Before the analysis, the sample was diluted in deionized water to form a 1% solution, and the volume of 50 µL was maintained constantly during the injection. Pullulan was a standard compound with concentrations from 0.342 kDa to 803 kDa. The MW of gelatin was calculated based on a pullulan standard curve. 

### 3.5. Antioxidant Activity 

The DPPH scavenging capacity of fish gelatin and SW-modified gelatin hydrolysates was analyzed by mixing 0.2 mL of samples with 0.8 mL of 0.1 mM DPPH. After vortexing for a few seconds, the mixture was allowed to react in the darkness for 30 min. The reacting mixture was then measured at 517nm using a multi-mode reader Synergy HTX. The blank was carried out similarly, except 0.2 mL of methanol was used instead of samples. The experiment was conducted in triplicate.

% DPPH scavenging = [1 − (A_o_ − A_s_)/A_o_] × 100.

A_o_ and A_s_ are the absorbance of the blank and samples, respectively. 

FRAP assay was conducted according to our previous study [32]. FRAP solution is a mixture of 300 mM acetate buffer (pH 3.6), 10 mM TPTZ in 40 mM HCl solution, and 20 mM FeCl_3_·6H_2_O solution (in a 10:1:1 ratio). There, TPTZ and FeCl_3_·6H_2_O solutions were freshly prepared. FRAP solution was incubated at 37 °C before use. Then, gelatin hydrolysates (0.75 µL) were mixed with 1.425 µL of the incubated solution, and the mixture was placed in the dark for 30 min. The reacting mixture was measured at 593 nm. A standard curve from 25 to 800 µM of Trolox was established, and the results were expressed as µM Trolox equivalent per gram sample. 

### 3.6. Preparation of Gelatin–Fucoidan Microspheres

The gelatin–fucoidan microspheres were prepared using a PL-micromed company’s technique. Gelatin-based microspheres, namely HG, SHG1, SHG2, and SHG3, consisted of fish gelatin and fucoidan or a mixture of fish gelatin, fucoidan, and SW-modified gelatin at 120 °C, 140 °C, and 160 °C, respectively (Table 3). A control (microspheres made of only fish gelatin) was also prepared in the same manner to compare surface morphology and drug-binding efficiency. 

### 3.7. Determination of Crosslinking Degree

Cross-linking degree of the cross-linked and uncross-linked microspheres was determined using a Trinitrobenzene sulfonate (TNBS) assay [29] with some modification. Briefly, microspheres (5mg) were placed in a test tube and mixed with 1 mL of 4% NaHCO_3_ for 30 min at room temperature. Then, 1 mL of 0.5% TNBS solution was added, and the mixture was immediately incubated at 40 °C for 2 h. After the incubation, 3 mL of 6N HCl was added into the incubated mixture to terminate the reaction, followed by another incubation at 60 °C for 90 min. Finally, 5 mL of distilled water was added to the resulting mixture and cooled to room temperature. The absorbance of the reactant mixture was measured at 345 nm using a microplate reader.

### 3.8. Swelling Property 

An exact amount of microspheres (50 ± 0.2 mg) was soaked in phosphate buffer saline pH 7.4 for 1H at 37 °C. Then, the swollen microspheres were filtered and were allowed to contact tissue that was put under the filter paper to remove the excess water on their surface for similar periods to minimize error. The experiment was repeated three times, and an average value was obtained. 

### 3.9. Fourier-Transform Infrared Spectroscopy 

FTIR spectra were obtained by measuring the spectra of KBr pellets containing approximately 2 mg of prepared microspheres and 250 mg of KBr using a spectrophotometer (Perkin Elmer, Shelton, CT, USA) with a range of 500–4000 cm^−1^. 

### 3.10. Morphology Analysis (SEM)

Surface characteristics of the microspheres were observed using a JSM-6490LC scanning electron microscope (JEOL, Tokyo, Japan). Before the analysis, the dried microspheres were carefully mounted onto the brass stubs and coated with gold in an argon atmosphere. 

### 3.11. Enzymatic Degradation 

Microspheres (50 mg) were soaked in 1.8 mL of 0.1% pepsin in 0.1N HCl. The mixtures were incubated at 37 °C in a water bath heated by a magnetic stirrer. The degradation process was observed and reported after intervals of time. The microspheres were then washed with distilled water before drying at 105 °C until their weight was unchanged. The ratio between the weight of dried residue to the initial weights is considered as its weight loss (%). 

### 3.12. Doxorubicin Absorption

Microspheres (10 ± 2 mg) were dispersed in 1 mL of 1 mg/mL Doxorubicin solution in distilled water at 37 °C for 1 h. The drug loading efficiency of microspheres was determined by measuring the concentration of the drug in the supernatant at 490 nm using a microplate reader. A standard curve of Dox from 0.005 mg to 0.4 mg/mL was established. The experiment was repeated three times.

### 3.13. Statistical Analysis

One-way analysis and Duncan test at a significance of 0.05 were conducted using IBM^®^ SPSS Statistics for Windows (Version 20.0., IBM Corp., Armonk, NY, USA). 

## 4. Conclusions

Gelatin with a high MW is not suitable for producing short-term transient embolic agents since it has long-term degradation in the human body. Therefore, in the present study, SW, which is considered a ‘green’ technique, was employed to reduce the MW of the protein. Due to the decrease in MW, changes in antioxidant activity and viscosity of SW-modified gelatin hydrolysates were observed. The dark color in SW-modified gelatin at 160 °C may be explained by the Maillard reaction rather than the thermal decomposition of protein molecules. Interestingly, SHG2, made of fish gelatin, fucoidan, and SW-modified gelatin at 120 °C, absorbed the highest amount of Doxorubicin among the microspheres. The most important finding might be the short-term enzymatic degradation of the microspheres made of SW-modified gelatin, although all microspheres were cross-linked at the same level. Cross-linking bonds between gelatin molecules are weaker than their intramolecular bonds. This might be one of the reasons for the phenomenon. The results indicated that SW could be a promising method to reduce the MW of biopolymers. In addition, gelatin–fucoidan microspheres made of SW-modified gelatin with a controlled degradation rate showed potential to be candidates for short-term embolic agents.

## Figures and Tables

**Table 1:** Color, pH, and viscosity of SW-modified gelatin hydrolysates.

Temperature (°C)	L *	a *	b *	pH	Viscosity (cP)
Original gelatin	-	-	-	-	7.18 ± 0.11
120	74.87 ± 2.8	−0.8 ± 0.25	2.2 ± 0.24	6.36 ± 0.04	1.6 ± 0.04
140	72.3 ± 1.62	−0.9 ± 0.22	7.1 ± 0.67	6.25 ± 0.02	1.0 ± 0.03
160	37.9 ± 1.22	−0.85 ± 0.1	1.46 ± 0.2	6.3 ± 0.02	0.7 ± 0.07

L *, a *, and b * are coordinates of the CIELAB color system.

**Table 2:** Changes in MW of SW-modified gelatin compared to fish gelatin.

Samples	Mn (kDa)	Mw (kDa)	PDI
Fish gelatin	6.6	26	3.96
120	4.1	12.2	2.98
140	1.7	4.1	2.36
160	1.4	2.8	2.06

120—SW-modified gelatin hydrolysates at 120 °C, 140—SW-modified gelatin hydrolysates at 140 °C, and 160—SW-modified gelatin hydrolysates at 160 °C.

**Table 3:** Preparation of gelatin–fucoidan microspheres.

Compositions	Gelatin–Fucoidan Microspheres				
	Control	HG	SHG1	SHG2	SHG3
Fish gelatin (%)	100	100	50	50	50
Subcritical water-modified gelatin at 120 °C (%)	0	0	50	0	0
Subcritical water-modified gelatin at 140 °C (%)	0	0	0	50	0
Subcritical water-modified gelatin at 160 °C (%)	0	0	0	0	50
Fucoidan * (%, *w/v*)	0	0.25	0.25	0.25	0.25

‘*’ is the percentage of fucoidan in gelatin solution (*w/v*).

## Data Availability

Not new data were created.
